# Terrorism as Coalitional Predation: Explaining Definitional Ambiguities and Precautionary Responses

**DOI:** 10.1177/14747049241263995

**Published:** 2024-07-25

**Authors:** Michael Moncrieff

**Affiliations:** 1 Department of International Public Law & International Organization, 27212 University of Geneva, Geneva, Switzerland

**Keywords:** terrorism, definition, countermeasures, coalitional conflict, precautionary psychology, manipulation, mental template, threat perception

## Abstract

Terrorism continues to be an enigmatic and contested concept, lacking a universally accepted definition despite extensive scholarly debate. Lay intuitions, however, demonstrate a notable convergence in identifying acts as “terrorism” when specific situational features are present, such as indiscriminate violence and out-group perpetration. These features elicit predictable and robust precautionary responses, raising the question: Is there a unified and parsimonious explanation for these phenomena? It is hypothesized that a situational template exists in the human mind, the coalitional predation template (CPT), which evolved not to detect modern-day terrorism, *per se*, but to identify and respond to situations of predatory coalitional conflict. The paper examines the potential cues and mechanisms that constitute the psychological systems activated by such threats, suggesting that matching the input cues of the CPT triggers well-documented precautionary responses to terrorism. However, this cue-based system may not align neatly with contemporary threats, leading to disproportionate responses to some threats while underestimating others. The model also posits that interpretations of violence can vary due to incomplete cues and the social position of the evaluator, leading to public disagreements and inconsistencies in defining terrorism. Consequently, arriving at an unambiguous and widely accepted definition of terrorism may not be possible. The model presented may account for a range of phenomena, including the inclination towards attributing mental illness to particular violent incidents and the uncanny surface similarities between terrorism and war crimes. The findings have significant implications for both the theoretical understanding of terrorism and practical policy responses.

## Background

Terrorism, in many ways, is an enigma. While the phenomenon has received a great deal of attention from scholars, lawyers, policymakers, the media, and the wider public, there remains no widespread agreement on a definition of terrorism (Schmid, 2023). Weinberg et al. (2004) note that the term has eluded consensus because it is an “essentially contested concept” subject to interpretative flexibility and varying political agendas. However, the reason *why* such subjectivity surrounds “terrorism” is itself something to be explained. While some concepts in international law, such as genocide, are defined with relative clarity and acceptance (O’Brien, 2020), terrorism continues to escape such consensus. As Richards (2018, p. 15) notes, the profound challenges in conceptualizing terrorism have led some to question the value of the endeavor itself. This ambiguity is not restricted to scholarly debates but also permeates public discourse and government policy (Dolliver & Kearns, 2022; Lazreg, 2017; Schmid, 2011).

Despite such disagreement, the public appears to achieve some consensus on the situational features that constitute “terrorism.” When presented with vignettes, certain acts seem to be characterized, *more or less*, like “terrorism” (e.g., Huff & Kertzer, 2018). Particular situational factors and individual differences affect whether a situation is represented as such. For instance, indiscriminate acts of violence are more likely to be considered “terrorism” compared to discriminate violence (e.g., Huff & Kertzer, 2018). Furthermore, acts committed by perpetrators from outside one's coalition are more likely to be deemed terrorism than those by members of one's group (D’Orazio & Salehyan, 2018; Hase, 2023; Huff & Kertzer, 2018; Kantorowicz et al., 2023; Lindner, 2018). Such effects of identity on judgment are not limited to labeling but also apply to attributions of culpability. In-group perpetrators are more likely to be viewed as having a mental illness compared to out-group members who are believed to be motivated by malevolent intent (e.g., Noor et al., 2019). Indeed, when U.S. participants were asked to envision the traits and characteristics of a “terrorist,” “mass shooter,” and “lone wolf,” distinct representations emerged (Campbell-Obaid & Lacasse, 2023). Participants depicted the mass shooter and lone wolf as more likely to be depressed, sad, lonely, white, and U.S. citizens. In contrast, terrorists were ascribed more demonizing traits, characterized predominantly by their out-group status and ideological motivations. This begs the question, Why do *particular* features matter so much but not others? And why do opinions vary widely between individuals yet sometimes show surprising agreement?

A second related puzzle is why certain situational cues trigger precautionary responses, such as hypervigilance and anxiety, compared to other situations that are objectively more frequent and harmful (Mueller, 2005a, 2005b; Sunstein, 2003). For example, data shows that in the United States, attacks and fatalities from white domestic perpetrators significantly outnumber those perpetrated by domestic Muslim or foreign terrorists (Williams, 2017). Yet, research indicates that public reactions and demands for harsh countermeasures are considerably less intense toward white domestic perpetrators (e.g., Conrad et al., 2018; Elmasry & el-Nawawy, 2020; Garcia & Geva, 2016; Piazza, 2015). Seemingly unrelated social information also seems to influence the endorsement of certain countermeasures. When participants are informed their coalition will soon lose its dominant status, they intensify their endorsement of harsher counterterrorism measures (Piazza, 2023). Anxiety also tends to persist for long periods of time after “terrorist” attacks. For instance, a poll among Americans spanning two decades found that fear of dying from terrorism has remained mostly consistent since 2001—approximately 68% worry a “fair amount” or “great deal” about the possibility of terrorist attacks (*Terrorism Poll*, 2021). Mueller and Stewart (2019, p. 2) note how such persistent fear has led to enormous resource allocations to counter a threat that “scarcely presents enough of a hazard to justify the enormous efforts and expenditures that have been made to counter it.” Indeed, many responses to terrorism appear economically and objectively unwarranted (Mueller, 2005a, 2005b; Sunstein, 2003). For instance, to be considered cost-effective, increases in American homeland security expenditures since 2001 would have had to prevent four large-scale attacks on U.S. cities each day (Mueller & Stewart, 2011, p. 11). How can we make sense of the *ambiguity* of the terrorism concept and the systematic *precautionary* responses we have toward *particular* acts of violence but not others?

This paper presents a novel hypothesis that definitional ambiguities of terrorism and responses to situations of mass violence can be accounted for by a situational template in the human mind (Barrett, 2014; Sperber, 1994) that identifies and responds to situations of all-or-nothing predatory coalitional^1^ warfare (Sell & Lopez, 2020). Importantly, this “coalitional predation template”^2^ (CPT) was not shaped to detect and respond to instances of modern-day terrorism. As such, many situations of modern-day mass violence may fail to align with the cues our psychological mechanisms evolved to recognize, which contributes to inconsistencies in how “terrorism” is represented and responded to. It will be shown that lay perceptions of “terrorism” align with the hypothesized inputs of the CPT. The manuscript situates the CPT as part of the “coalitional safety system,” an internal regulatory system that detects and responds to coalitional threats (Boyer et al., 2015). This functional system helps make sense of the systematic precautionary responses to terrorism found in the literature.

This paper builds upon previous scholarship that approaches terrorism through the lens of evolutionary psychology and coalitional threat assessments (Kiper & Sosis, 2015; Lindner, 2018). It is important to note that this paper does not seek to engage with normative questions of what responses to terrorism *ought to be*. Nor does it seek to debate the effectiveness of measures that help prevent and mitigate mass violence (for insightful discussions, see Mueller & Stewart, 2011, 2016). Instead, it aims to account for *why* specific cues associated with certain situations of violence systematically elicit more pronounced responses than situations lacking such cues.

The subsequent sections will review research on situational threat templates and the coalitional safety system, focusing on an analysis of hypothesized input cues that might activate the CPT and the downstream effects of its activation. The paper concludes with an examination of the broader implications of the model, particularly regarding conceptualizations of terrorism.

## Detecting and Responding to Coalitional Threats

Empirical evidence suggests that aggressive collective conflict among our ancestors was substantial enough to exert significant selection pressure (Keeley, 1996; Tooby & Cosmides, 2010). This pressure has shaped the human mind with a suite of psychological mechanisms, referred to as coalitional psychology. These mechanisms are specialized for reasoning about coalitional dynamics, detecting threats, and motivating specific actions (Boyer et al., 2015; Cikara, 2021; Ermer, 2007; Kurzban et al., 2001; Kurzban & Neuberg, 2005; Lopez, 2017; Pietraszewski, 2016, 2022; Tooby & Cosmides, 2010; Winegard et al., 2020), which are crucial for navigating the complexities of multi-agent conflicts (Pietraszewski, 2016). Furthermore, they reflect the statistical regularities of the ancestral coalitional landscape, including recurrent forms of coalitional violence (Lopez, 2017).

### Evolved Mechanisms for Threat Detection

The human mind evolved to respond effectively to recurrent and potent threats during our evolutionary history (Neel et al., 2020). Situations such as intergroup conflict had significant implications for survival and reproductive fitness (Tooby & Cosmides, 2010), necessitating rapid and effective psychological responses. Responses to recurrent threats are often governed by situational mental templates that utilize detectable cues—information reliably associated with specific threats across generations (Barrett, 2005, 2014). Such templates—“sets of prepared cues, complexly combined” (Barrett, 2014, p. 234)—enable rapid identification and reaction to threats, thus enhancing fitness by reducing the risk of injury or death (Griffin et al., 2001; Nesse, 2005; New & German, 2015; Rakison, 2018; Rakison & Derringer, 2008).

Functioning as inference systems, mental templates process input cues to generate mental representations (Mercier & Sperber, 2017). Subsequent inferential procedures interact with representations to modify, erase, or refine them (Mercier & Sperber, 2017). Representations may not fully capture the complexity of the input or might only partially activate downstream procedures, depending on the completeness of the cues. For instance, the conceptualization of a “group” may differ between individuals based on their interpretation of cues and social position (Pietraszewski, 2022). Similarly, interpretations of situations of violence may vary as the presence of incomplete cues, coupled with self-interest, can contribute to inconsistencies in how people represent violence (e.g., “it kind of looks like terrorism”). As discussed in subsequent sections, individual differences (e.g., coalitional membership, risk exposure) may cause people to represent the same situation differently (Neel et al., 2020). Such variability affects how individuals coordinate on public representations such as “terrorism,” potentially leading to disagreements over its definition and what constitutes it.

Evidence suggests that threat detection systems are shaped by error management biases that favor false alarms over missed threats, akin to a sensitive smoke alarm that errs on the side of caution (Haselton & Buss, 2000; Nesse, 2001, 2005; Nesse & Williams, 1996). Indeed, some threats with low probabilities but potentially detrimental effects favor the deployment of countermeasures even when error rates are high (Szechtman & Woody, 2004; Tooby & Cosmides, 2006; Woody & Szechtman, 2011). For the model presented here, this implies that specific cues indicative of inter-coalitional threats may evoke a higher rate of false-positive errors in evaluative and precautionary responses (Lindner, 2019). Consequently, there may be a tendency to perceive existential coalitional threats even when the enemy is too weak to pose a significant threat. This tendency to overreact to potentially extreme situations ensures that the most significant potential outcomes—those with extreme utilities—are always taken into consideration (Lieder et al., 2018).

Although finely tuned to detect specific threats, detection systems are also inherently susceptible to “cognitive capture,” in which the system reacts to stimuli beyond its intended functional scope (Sperber, 1994; Sperber & Hirschfeld, 2007). This is an important point, for it is argued that the “proper domain” of the CPT includes cues associated with predatory coalitional conflict, but the “actual domain” might include cues generated by other forms of modern-day aggression (e.g., “terrorism”). Indeed, the great uncertainty in detecting and responding to threats makes such systems “susceptible to error, volatile reweighting, individual differences, and social entrainment (including manipulation)” (Tooby & Cosmides, 2006). Once activated, precautionary responses to threats, such as hypervigilance and anxiety, may persist, especially when the absence of cues does not imply the absence of the threat (Hinds et al., 2010; Szechtman & Woody, 2004; Woody & Szechtman, 2011). In this regard, cues indicating safety (Luttbeg et al., 2020) and the completion of actions perceived as necessary to mitigate the threat (Boyer & Liénard, 2006; Hinds et al., 2010; Woody & Szechtman, 2011) might be necessary for lowering or terminating the activation of precautionary responses. Such long-term activation of precautionary responses might explain the persistence of terrorism anxiety noted earlier (Terrorism Poll, 2021).

## The CPT within the Coalitional Safety System

Central to responding to coalitional threats is the coalitional safety system (Boyer et al., 2015). Within this system, Boyer et al. (2015, p. 438) hypothesize the existence of the coalitional safety index (CSI), an internal regulatory variable that integrates cues from the individual's social environment, including perceived threats outside one's coalition and available support from within. This index is similar to other regulatory mechanisms that integrate internal and external informational inputs to guide adaptive behavior (Cosmides & Tooby, 2000; Tooby et al., 2008; Tooby & Cosmides, 2008). Functionally, the CSI adjusts an individual's perceived safety within a coalitional context by monitoring and responding to changes in inter-coalitional dynamics. When the CSI detects a decrease in intra-group support or an increase in intergroup threat, it triggers adaptive responses designed to restore safety. Lowering the CSI leads to the activation of precautionary plans motivating behaviors aimed at mitigating the threat and restoring a higher level of coalitional safety. Such plans may include the reinforcing of in-group homogeneity and cooperation, avoiding members of other alliances, and engaging in competition or conflict with rival coalitions (Boyer et al., 2015; Gneezy & Fessler, 2012; Puurtinen & Mappes, 2009).

Given what is known about situational threat templates and the CSI, it is hypothesized that a situational template exists in the human mind specifically tuned to respond to the most lethal form of collective aggression—predatory coalitional conflict. Predatory coalitional conflict, characterized by uncompromising lethal aggression aimed at the destruction of rivals, is likely to have been a significant evolutionary pressure shaping human coalitional psychology (Sell & Lopez, 2020). Thus, the CPT should be tailored to recognize and react to situations of coalitional conflict where the primary objective is the elimination of the enemy. The CPT builds upon the coalitional safety system by elucidating some of the specific cues that may be used by the system to detect situations of predatory coalitional conflict and their downstream effects on precautionary responses.

To function effectively, the CPT should process cues related to two summary variables of coalitional threats. First, it must assess the formability and capability of potential adversaries to inflict harm (“cost infliction capabilities”), as evidenced, for instance, by the number of agents and their cohesiveness—attributes that reflect their capacity for coordinated action and potential to inflict damage (e.g., Boyer et al., 2015; Fessler & Holbrook, 2016). Second, it should evaluate cues that indicate the adversaries’ dispositions (“shared intentional stance”) (Pietraszewski, 2016), specifically indicators of a predatory stance toward one's own coalition (Sell & Lopez, 2020). This dual focus on capability and intention aligns with broader psychological theories positing that other agents’ *disposition* to help or harm and *capability* to act accordingly are core components in assessing social threats (Fiske, 2018; Hack et al., 2013). Fessler and Holbrook (2016) further suggest that evaluations of coalitional cohesion and formidability involve aggregating various relevant cues into a coherent assessment that aids coalitional decision-making. The same can be said of evaluations of aggressive dispositions (Scrivner et al., 2020). While a comprehensive understanding of the full range of cues integral to the coalitional safety system remains an open area for research, some scholars have begun to highlight probable candidates (e.g., Boyer et al., 2015; Fessler & Holbrook, 2016; Lindner, 2018; Pietraszewski, 2016, 2022; Sell et al., 2024; Sell & Lopez, 2020).

**Figure 1.**
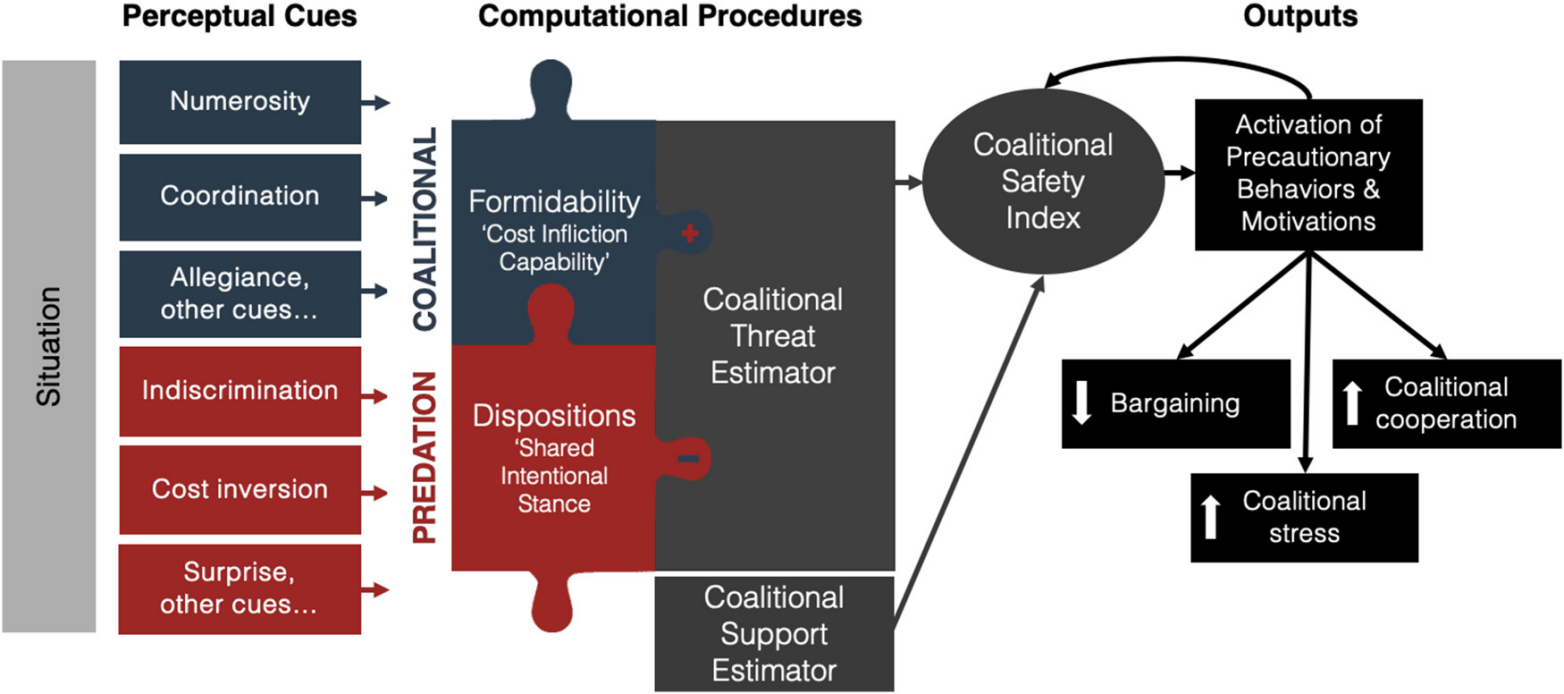
A schematic representation of the coalitional safety system. This figure illustrates the information-processing structure of the coalitional safety system, adapted from Boyer et al. (2015). It illustrates how cues related to “cost infliction capabilities” (the “coalitional” element of the template) and “shared intentional stance.” (the “predation” element of the template) are processed. Cues such as numerosity, coordination, and indiscrimination inform the computational procedures, which estimate the summary variables “Formidability” and “Dispositions.” These estimations are integrated by the Coalitional Threat Estimator, whose outputs are further integrated with the Coalitional Support Estimator (cues not shown). The combined estimates adjust the CSI positively or negatively. Negative adjustments of the CSI trigger precautionary responses, such as increased coalitional cooperation and stress, among other cognitive and behavioral changes.

Figure 1 integrates some of the hypothesized sets of cues and summary variables into the broader CSI model (Boyer et al., 2015) by illustrating how the CPT assesses cues indicating (1) cost infliction capabilities and (2) shared intentional stance. This expanded model aids in understanding how various cues are aggregated into a coherent assessment that informs decision-making and precautionary responses in situations of coalitional conflict.

The concept of the CPT expands upon previous scholarship, including the work of Kiper and Sosis (2015) and Lindner (2018). Kiper and Sosis (2015) discuss the evolutionary psychology of precautionary responses to terrorism, proposing a “terror module” that responds to extremely threatening stimuli such as signs of death or violence—a somewhat broader account of the psychological effects of terrorism that does not directly address the relevance of coalitional threat cues. This paper aligns with Kiper and Sosis’s (2015; 103) perspective that “terrorism must exploit a psychological system dedicated to extreme threats and uncertainties.” However, the approach here differs in how to account for the specifics of that system. Lindner (2018), on the other hand, discusses how coalitional psychology shapes public reactions to terrorism, focusing on the adaptive challenge of coalitional aggression and between-coalition threat assessments. In comparison, the current paper posits the involvement of the CPT and hypothesizes that human minds were evolutionarily tailored to respond to *particular cues* associated with predatory coalitional conflict rather than modern-day terrorism. Importantly, as discussed later, the CPT may account for a range of effects noted in the terrorism literature, including the systematic attribution of mental illness to particular attacks over others (e.g., Noor et al., 2019) and noted similarities between terrorism and war crimes (Schmid, 1992), aspects not thoroughly addressed in previous scholarship.

The next sections will detail the cues likely to be most relevant to the summary variables “cost infliction capabilities” and “shared intentional stance.” A concurrent review of the terrorism literature shows that the cues used to evaluate situations of violence align with the CPT's hypothesized input cues. Furthermore, once the hypothesized cues activate the CPT, its activation triggers downstream effects that are parsimoniously accounted for by the coalitional safety system.

### Assessing Threat: Coalitional Formidability Cues

Coalitions are not objectively real but rather exist to the extent that they are consistently perceived and understood in the minds of various individuals (Tooby et al., 2006). As such, a coalition only becomes threatening when a certain set of agents behave similarly with regard to the delivery and exchange of costs toward some other agents (Pietraszewski, 2019). For example, if agent A attacks agent D and subsequently is defeated by D, the threat of the attack ends. However, if agents B and C also behave similarly as A did to D, the threat of further attacks still exists. Knowing that B and C still exist, D should be concerned about the likelihood of future harm. The summary mental representation of a “group” is constituted, in part, by the unfolding of contingent cost/benefit interactions (Pietraszewski, 2016, 2022).

This logic likely underlies the distinction made between coalitional (“terrorist”) versus non-coalitional aggression (“mass shooter,” “lone wolf”) when it comes to public representations of violent acts (Lindner, 2018). Lone attackers, who lack others inclined to emulate their actions, do not pose the same sustained threat as perpetrators of coalitional violence. Thus, such threat perception is fundamentally rooted in the implication that others may harbor similar intentions and pose a continuing threat. This concern is not solely about an individual's coalitional membership but rather the broader implication that such membership suggests a wider network of agents who may exhibit similar hostile behaviors. This understanding is critical in differentiating between perceptions of isolated threats and those connected to a larger, potentially hostile coalition. D’Orazio and Salehyan (2018) illuminate this distinction, explaining how “lone wolves”—attackers who lack direct coalitional support—“may be dismissed as a single, isolated threat, while ties to a larger organization imply that others hold similar, extreme beliefs.” This also explains why, compared to individuals or small groups, attacks carried out by organizations, especially those with international connections, or by members of widely recognized coalitions, such as religious or political groups, are more likely to be viewed as acts of terrorism by the public (Huff & Kertzer, 2018) and reported as such by the media (Betus et al., 2021). The coalitional dimension also shows up in the many rigorous attempts to define terrorism by academics. For instance, Saul (2006, p. 59) defined terrorism based on a deductive analysis of existing international law and included, as a central component, that the act need be “for a political, ideological, religious, or ethnic purpose […].”^3^ The pervasive association of terrorism with coalitional features reflects one core component of the CPT.

At times, shared properties such as social identity features or coalitional markers may lead attackers to treat other agents as substitutable with one another (Pietraszewski, 2016). For instance, an attack targeting individuals with a common social identity impacts not only those directly attacked but also others similarly identified (e.g., Goode & Smith, 2016). Reactions to violence in a public space can shift dramatically from alarm to relief upon realizing the violence was targeted (e.g., gang-related) rather than indiscriminate and random, resulting in a lessened perceived threat to the general population.

It is important to note that the reality of group dynamics reveals that there is not a singular group or alliance; individuals belong to multiple, context-dependent nested alliances. This complexity suggests that the more imbricated the coalitional identities, the more likely individuals are to favor negotiation over aggression. Indeed, fitness interdependence typically limits the advantages of aggression within cooperative networks, while individuals outside these groups are more likely to be perceived as fitness suppressors (Aktipis et al., 2018; Tooby & Cosmides, 2010). The mere expectation of future interdependent interactions can enhance cooperative behavior (Blake et al., 2015; Van Lange et al., 2011; Wolf et al., 2009), thus influencing attitudes toward perpetrators. Indeed, the anticipation of continued interdependence also affects punitive behaviors. Individuals are more likely to administer punishment when future interactions are expected, using such measures as a deterrent against non-cooperative behavior (Krasnow et al., 2012). Such coalitional dynamics can significantly affect the persistence and nature of the threats posed by other coalitions.

#### Formidability Cues and Terrorism Perceptions

The impact of identity features on decision-making is clearly reflected in the terrorism literature. Attacks committed by out-group members who share few overlapping coalitional identity features, such as Jihadist extremists, are more frequently labeled as terrorists by those in the United States compared to actions by in-group factions like right or left-wing extremists (Fishman & Marvin, 2003; Hase, 2023; Kearns et al., 2019; Sui et al., 2017). A robust and consistent finding among terrorism studies is that acts of violence committed by actors external to one's coalition are more likely to be labeled as terrorism (D’Orazio & Salehyan, 2018; Hase, 2023; Huff & Kertzer, 2018; Kantorowicz et al., 2023; Lindner, 2018). Indeed, experimental research indicates that crossing coalitional boundaries is a central factor in determining if an act of violence is labeled as terrorism (Lindner, 2018). The concept of “group-constitutive roles” (Pietraszewski, 2016, 2022) may help operationalize and clarify what it means to “cross coalitional boundaries.”

Assessing the risk a coalition poses requires considering additional information, such as the number of agents and their cohesiveness (Boyer et al., 2015). A larger coalition amplifies the threat as its strength and potential for sustained aggression increase proportionally with its numbers (Benenson et al., 2009). The efficacy of a coalition lies not only in its size but also in its members’ ability to coordinate and exchange resources efficiently (Hardin, 1995). Indicators of a coalition's potential to cause harm, including its size, cohesiveness, and member dedication, should be essential for assessing its threat level (Boyer et al., 2015). Importantly, however, direct evidence of these factors is often elusive. In such situations, indirect cues, such as the group's coordination capabilities, may provide insights into its formidability (Fessler & Holbrook, 2016; Holbrook & Fessler, 2013). These insights extend into the terrorism literature, where cues of coalitional cohesiveness and formidability significantly increase the likelihood of acts being labeled terrorism. For instance, events demonstrating coordinational sophistication are perceived as more threatening because they indicate a greater capability to inflict harm (Avdan & Webb, 2018).

Given the difficulty in directly observing many cues of a coalition's capabilities, such as actual membership numbers, there is a tendency to overestimate rather than underestimate risk. This bias likely contributes to the inflated perceptions of terrorist groups like al-Qaeda. Despite common beliefs about their extensive network, al-Qaeda's core membership in 2002 was only 170 (Blair, 2012), with intelligence estimates rarely exceeding 500 members in other years (Mueller & Stewart, 2010). Additionally, Kenney (2009) highlights the lack of operational sophistication among many Jihadist militants, who frequently make errors and demonstrate poor planning skills. This ineptitude, along with significant coordination and communication flaws within terrorist networks (Eilstrup-Sangiovanni & Jones, 2008), challenges the perception of these groups as existential threats. Mueller and Stewart argue (2010, p. 6) the idea of such groups posing “an existential threat to just about anybody seems at least as fanciful as some of their schemes.”

### Assessing Threat: Coalitional Dispositional Cues

In addition to a coalition's capabilities to inflict harm, understanding the motivations of those agents is crucial for determining the degree of threat it poses. Sell and Lopez (2020) argued that the emotions of anger and hatred have distinct cost/benefit logics, which are reflected in the character of collective violence (e.g., the selection of targets, the character of escalation, and the possibility of surrender and reconciliation). As such, specific characteristics of how collective violence is conducted may serve as cues that indicate the motivations underlying coalitional aggression. Anger motivates bargaining behavior—the imposition of costs on others to get them to treat you better in the future or to cede resources.^4^ Anger-based aggression involves low-cost, attention-getting, formidability-enhancement displays, rule-based escalation, and the cessation of aggression once concessions have been met (Sell & Lopez, 2020). In comparison, hatred motivates actions that depower or eliminate others whose existence is perceived to impact your fitness negatively (Sell et al., 2024). Hate-based collective aggression is predatory in nature, characterized by a lack of communication before an attack, the infliction of high costs when the target is vulnerable, and increased aggression when the target signals weakness. As anger and hatred are distinct emotions, they can individually or jointly influence the nature of conflict.

When facing a situation of bargaining aggression, numerous opportunities exist to assess and respond to the threat. This type of warfare often involves significant “ritualization” (e.g., Chagnon, 1983; Sell, 2011; Sell & Lopez, 2020), where cues of coalitional formidability are abundant and frequently generated, whether honestly or deceptively. In contrast, predatory coalitional aggression lacks signals and occurs by surprise and without warning, necessitating rapid and prepared responses. Importantly, there is no chance to reassess a misjudged situation involving predatory aggression*.* Furthermore, as the objective of predatory aggression is to eliminate the enemy, signs of weakness or vulnerability reduce the perceived cost of attack, thereby increasing aggression when attempts to bargain are made. Thus, the optimal countermeasures are to either match the intensity of the aggression (retaliation) or flee the situation (fear). Consequently, the countermeasures shaped by predatory aggression are likely distinct from those of bargaining aggression, leading to specific downstream responses, some of which may be evident in responses to terrorism (see section “Precautionary Outputs of the Coalitional Safety System”).

#### Dispositional Cues and Terrorist Behavior

The distinction between anger and hate-driven aggression aligns with Schmid's (1992) conceptualization of terrorism. Schmid's work implicitly recognizes the presence of predatory aggression in terrorism, which mirrors the characteristics of total warfare due to its inherently predatory nature. In such a context, norms and moral restraint are less likely to develop as the primary goal is enemy annihilation. This insight supports Schmid's (1992) analogy of terrorism as “the peacetime equivalent of war crimes,” a comparison that resonates with the predatory character of total warfare. Mirroring the aggressive, norm-eschewing nature of total warfare, terrorism reflects the input conditions the CPT evolved to detect, thus rendering its comparison to war crimes particularly apt.

Widespread consensus on labeling attacks as terrorism requires more than just coalitional cues; it requires additional information clarifying the motivation of the attackers. When an attack indicates the goal is to eliminate rather than negotiate, it should activate the CPT. Such notions of unrestrained and indiscriminate aggression show up in Schmid's (2012) revised academic consensus definition of terrorism: “violent action without legal or moral restraints, targeting mainly civilians and non-combatants […].” The tactical choices of terrorists are often interpreted as indicators of their underlying motivations. Abrahms's (2006, 2013) demonstrates that the selection of civilians as targets is often perceived as evidence of the terrorists’ pursuit of uncompromising, maximalist objectives. Indiscriminate violence also heightens the perception that attacker coalitions represent a more severe threat to the societies they attack (Avdan & Webb, 2018). This makes sense as indiscriminate attacks strongly imply the nonrestraint that characterizes hate-based aggression (Sell & Lopez, 2020). In contrast, the attacking of military targets more closely resembles bargaining aggression. While bargaining aggression may coincide with predatory aggression, acts motivated entirely by bargaining aggression should lack the predatory cues necessary to activate the CPT.

#### Understanding “Irrationality” in Terrorism

Extreme acts of violence, often deemed by observers as “irrational,” challenge traditional conceptions of rationality due to their high costs and ostensibly low rewards for the perpetrators. Living in relatively peaceful societies organized around *cooperative rationality* equips us with goals, desires, and strategic sensibilities appropriate to the cooperative social ecology in which we live (Tooby, 2015). This, in turn, shapes how we interpret others’ intentions. Tooby (2015) explains that individuals operating under *predatory rationality* adopt a mindset centered on self-supremacy and engage in conflict with a zero-sum perspective, which stands in stark contrast to the mutual-benefit orientation inherent in cooperative rationality. When extreme emotions like hate motivate violence, perpetrators are guided by the logic of predatory rationality. Hatred can reorient perceived costs imposed on others, often severe and violent, into perceived benefits for the perpetrator (Sell et al., 2024; Sell & Lopez, 2020). Many acts of violent extremism exemplify this cognitive inversion. Motivated by emotions such as envy (Moncrieff & Lienard, 2023), perpetrators perceive the infliction of harm to others as beneficial and emotionally rewarding (Elbert et al., 2016; Nell, 2006). Such “cost inversion” may also account for why gruesome acts of violence increase the perception of perpetrator threat characteristics such as formidability (Scrivner et al., 2020). Their violent acts reveal an altered motivational state that, while appearing “irrational” to those operating under the logic of cooperative rationality, aligns with the perpetrator's subjective predatory rationality.

Empirical evidence supports the logic presented here. Violent acts perpetrated by fellow coalition members are more likely to be explained by attributing the behavior to mental illness,^5^ *i.e., irrationality* (e.g., Betus et al., 2021; Ghazi-Tehrani & Kearns, 2020; Huff & Kertzer, 2018; Kunst et al., 2018; Noor et al., 2019). On the other hand, when cues suggest the perpetrator is a member of an opposing coalition, recognizing the predatory motivations causing the violence comes more intuitively.^6^ Furthermore, acts involving a higher likelihood of indiscriminate harm (e.g., bombings) and greater extremity of violence (e.g., violence versus protest) are indeed more likely to be labeled as terrorism (Huff & Kertzer, 2018), matching the hypothesized cues of the CPT. Experiments demonstrate that participants focus on attack severity (number of casualties) over the probability of an attack when deciding how to allocate counterterrorism resources (Kantorowicz et al., 2023). Highly lethal attacks in Western countries are also more likely to be labeled as terrorism by the press (Ghazi-Tehrani & Kearns, 2020; Hase, 2023; Nagar, 2010). Finally, the element of surprise and the attacking of unprotected “soft” targets, both characteristics of predatory aggression, are also key factors influencing whether an attack is viewed as terrorism (e.g., Sinai, 2008). Indeed, a lack of communication and surprise characterizes predatory attacks aimed to inflict high costs when the target is vulnerable, accompanied by increased aggression when the target signals weakness (Sell & Lopez, 2020).

### Precautionary Outputs of the Coalitional Safety System

Appropriately responding to predatory coalitional conflict involves recognizing the predatory rationality of the perpetrators and countering it effectively. When modern-day terrorism emulates the input conditions the CPT is attuned to, activating it should result in a dramatic reduction in an individual's CSI (Boyer et al., 2015). All else being equal, such a dramatic reduction should trigger a range of countermeasures, including specific behaviors aimed at mitigating the perceived threat, a state of hypervigilance, and stress responses (Boyer et al., 2015; Boyer & Liénard, 2006; Gneezy & Fessler, 2012; Puurtinen & Mappes, 2009; Woody & Szechtman, 2011). Furthermore, De Dreu and Gross (2019) argue that defense should be associated with the activation of neural circuitries involved in behavioral inhibition. Accordingly, those threatened by a seemingly predatory coalition might report extremely negative feelings, such as fear, disgust, and resentment, and in cases of survival, relief (De Dreu & Gross, 2019). Emotions that organize the type and intensity of activated countermeasures against the threat.

When faced with an enemy group intent on eliminating one's coalition, the response should be characterized by heightened vigilance, aggressive defense postures, and a strong emphasis on coalitional cohesion and solidarity (e.g., Lopez, 2017; Moncrieff & Lienard, 2019; Navarrete et al., 2004; Schaller & Abeysinghe, 2006). Conversely, when the threat involves an opposing group employing bargaining aggression, the response might be less about survival and more about maintaining or negotiating social hierarchies and resources, resulting in more calculated and strategic responses (Sell & Lopez, 2020). When threats are perceived as existential, as with predatory aggression, they engage survival mechanisms that elicit strong retaliatory responses characterized by a comprehensive, all-hands-on-deck approach aimed at safeguarding the coalition (De Dreu & Gross, 2019). In the latter scenario, there may be an increased preference for dominant leadership as it is more conducive to coordinating aggression (Laustsen & Petersen, 2017).

#### Variability in Individual Responses to Terrorism

The concept of situational “potency” can be understood as the likelihood that a particular situation would have had significant reproductive fitness impacts in ancestral environments (Neel et al., 2020). It is intriguing to consider the variability in evolutionary conditions across human populations and how such variability influences modern responses to coalitional aggression (e.g., terrorist attacks).^7^ While such theorizing is beyond the scope of this paper, it is clear that cues of inter-coalitional conflict activate a wide range of inferential and motivational systems. The full scope of their effects has yet to be established and will require further study (Lopez, 2017). However, as situations of predatory aggression were more reproductively consequential than situations of bargaining aggression, we should expect more typified and robust responses to the former. For instance, as predatory aggression is indiscriminate and affects *all* members of the defending group, we should expect increased cooperative tendencies to be highly uniform (e.g., the “rally ‘round the flag” effect) (Godefroidt, 2023; Kuehnhanss et al., 2021). As bargaining aggression impacts certain individuals within a coalition with more or less opportunities and costs, we should witness greater variation in responses given individual differences (e.g., sex, age, body size, aggressivity, fighting ability).

Individual differences may lead individuals to respond differently to the same threat cues because the situation poses different adaptive problems or because the situation affords different response strategies (Neel et al., 2020). For example, while men might typically respond with anger and aggression toward enemy males, for women, the potential risks of physical violence and sexual assault or infanticide may make it more adaptive to exhibit fear and avoidance toward enemy males (McDonald et al., 2012). Indeed, exposure to terrorism is associated with negative health consequences for offspring (Quintana-Domeque & Ródenas-Serrano, 2017) and increased rates of male fetal loss (Bruckner et al., 2010). In response to terrorism, empirical data shows that males are more prone to experiencing retaliatory emotions (i.e., anger) but less fear than females; these emotions account for a large proportion of the differences in risk perceptions and predict diverging public policy preferences (Lerner et al., 2003).

#### Evidence of Precautionary Responses to Terrorism

If modern-day terrorism activates the CPT by emulating the input conditions it is attuned to, then we should expect pronounced responses to terrorism. In accordance with the CPT hypothesis, responses to acts of terrorist violence should manifest in psychological and behavioral countermeasures characterized by heightened vigilance, retaliatory aggression, and a pervasive sense of fear (for in-depth reviews, see Sinclair & Antonius (2012) and (2013)). Indeed, terrorist attacks elicit vengeance, hostility towards in-group detractors, widespread fear, anxiety, and a heightened desire for security (e.g., Conrad et al., 2018; Davis & Silver, 2004; Huddy et al., 2005; Lerner et al., 2003; Schuster et al., 2001; Sinclair & Antonius, 2012). Because countermeasures are often deployed against threats with low occurrence rates but potentially high impacts (Szechtman & Woody, 2004; Tooby & Cosmides, 2006; Woody & Szechtman, 2011), it makes sense why terrorism leads to distorted risk assessments and overestimations of terrorism's frequency and lethality (Kearns et al., 2021; Makkonen et al., 2020). Sunstein (2003) accounts for this effect using the concept of “probability neglect”—people react so strongly because they focus on the badness of the outcome while ignoring the low probability of the event (for a similar argument, see Gigerenzer (2006)). However, such accounts cannot explain why reactions to situations of violence such as terrorism appear sensitive to coalitional and predatory threat cues and not merely outcomes. As previously noted, the observation that anxiety and other precautionary responses may persist when cues do not signal the termination of the threat (Szechtman & Woody, 2004; Woody & Szechtman, 2011) aligns with data on terrorism fears. Despite the minuscule risk of being involved in terrorism, the fear of terrorist attacks is a long-enduring phenomenon (Misis et al., 2017; Sinclair & Antonius, 2012).

The body of research on responses to terrorism suggests that when acts of violence effectively trigger the CPT, there is a consistent and predictable shift in rationality and preferences. The perception that terrorists are irrational (indicative of a predatory mindset), especially those identified as out-group members, is linked to escalated fear, a greater sense of impending risk of future attacks, and a preference for aggressive countermeasures (i.e., military action) over diplomatic (i.e., bargaining) approaches (Kossowska et al., 2010; Pronin et al., 2006).

Following terrorist attacks, there are often abrupt changes in public support for aggressive policies and a propensity to prioritize collective security over individual autonomy (Echebarria-Echabe & Fernández-Guede, 2006; Huddy et al., 2009). However, acceptance of reductions in civil liberties is specific to transnational terror threats and not domestic terrorist threats, regardless of the level of domestic threats or the effectiveness of policy responses (Garcia & Geva, 2016). Other attitudinal shifts include increased support for aggressive retaliatory measures such as the torture of terrorism suspects (Conrad et al., 2018; Piazza, 2023), hawkish foreign policy measures (Gadarian, 2010; Garcia & Geva, 2016; Holman et al., 2016), and public opinion favoring hostile counterterrorism policies (Godefroidt, 2023; Huddy et al., 2005; Lerner et al., 2003). Notably, support for the torture of terrorism suspects and indefinite detention is predicated on cues of external coalitional membership (Arab descent, Muslims) combined with predatory intent (suspected “terrorist”) (Conrad et al., 2018; Piazza, 2015). Whereas there is less support for treating domestic, right-wing terrorist suspects with uncompromising measures, such as indefinite detention (Piazza, 2015). Individuals are also less inclined to support government negotiations when organizations display cues of predatory behavior (e.g., bombing) compared to bargaining aggression (e.g., demonstrations, occupations) (Huff & Kruszewska, 2016). Perceptions that terrorists are motivated by hatred are also positively correlated with the endorsement of harsher counterterrorism measures (Canetti et al., 2021). These natural responses align with the anticipated shift towards a retaliatory posture that seeks to counteract the predatory rationality of attackers.

The psychological impact of terrorism extends beyond direct victims, as indirect exposure through the media may also trigger significant psychological responses (Danieli et al., 2004; Miguel-Tobal et al., 2006; Rubin et al., 2007; Schlenger et al., 2002; Sinclair & Antonius, 2012). Such exposure can lead to a surge in support for assertive security policies and the strengthening of in-group cohesion and solidarity, known as the “rally ‘round the flag” effect (Chowanietz, 2011; Geys & Qari, 2017; Godefroidt, 2023; Huddy & Feldman, 2011; Kuehnhanss et al., 2021). The activation of particularly precautionary psychological systems also seems to affect policymakers. Woody and Szechtman (2013) hypothesize that the segmentation of roles within security organizations might impair the stopping mechanism of precautionary responses. Policymakers not engaged in directly implementing protective actions may lack the feedback needed to satisfy the system's need for action, so no degree of precaution feels sufficient. Precautionary psychological responses could also be responsible for the increasing creep of administrative “pre-crime” counterterrorism measures (see Global Study on the Impact of Counter-Terrorism Measures on Civil Society and Civic Space, 2023), as the push for successful prevention aims to preclude such adverse events altogether (Tooby & Cosmides, 2006).

In sum, the range of responses to terrorist threats, from heightened fear and anxiety to support for aggressive and preventative policies, matches what would be expected if cues associated with terrorism align with the CPT, thus activating precautionary psychological responses.

## Discussion

What are we to make of “terrorism”? More profoundly and pertinently, “How are we to understand minds that take joy in slaughtering the innocent?” (Tooby, 2015). The model presented in this article fills a gap in the literature by offering a unified explanation for various terrorism-related phenomena. It explains why certain features of mass violence are more salient than others, why definitional ambiguities abound, and why specific systemic downstream effects are triggered by particular sets of features.

The existing evidence aligns with the notion that certain contemporary acts of mass violence activate the hypothesized CPT by matching the input conditions it evolved to respond to. This activation explains the systematic precautionary responses observed in reaction to “terrorism” as the CPT engages the coalitional safety system (Boyer et al., 2015). While many scholars contend that the efficacy of terrorism is rooted in its ability to evoke fear, the underlying mechanisms of *why* such fear is evoked have remained less examined (however, see Gigerenzer, 2006; Kiper & Sosis, 2015; Lindner, 2018; Sinclair & Antonius, 2012; Sunstein, 2003). The insights gleaned from this model afford new perspectives on a range of phenomena shaped by perceptions of and responses to terrorism. The model also sheds light on how terrorist tactics influence broader societal reactions and policy responses to terrorism.

### Implications of the CPT

If the CPT hypothesis is correct, it affects how we define, understand, and respond to terrorism. It suggests that a universally accepted definition of terrorism may be unattainable because many situations of modern violence do not fully align with the specific set of conditions our psychological mechanisms were shaped to recognize and respond to. Additionally, individual differences may cause people to represent the same situation differently (Neel et al., 2020). These differences significantly contribute to the lack of coordination on a shared public representation of “terrorism,” fundamentally underpinning the persistent challenges in achieving a widely accepted conceptualization of terrorism (regarding its ontology, see Jackson (2008)).

There is general agreement that terrorism is “a violent communication strategy for psychological (mass) manipulation” (Schmid, 2023, p. 14). However, if the model here is correct, we should question the assumption that the goal of terrorism, *in all instances*, is to manipulate governments and the public into ceding political concessions (e.g., Atkinson et al., 1987). As predatory aggression aims to eliminate or significantly depower adversaries (Sell & Lopez, 2020), it is unclear why such predatory tactics would be appropriate if the goal of such aggression is to bargain politically (also, see Abrahms, 2011). This may be the reason why terrorist groups are generally *less likely* to achieve their supposed political objectives than non-terrorist groups (Abrahms, 2006; Fortna, 2015). Essentially, terrorism generally does not “work” because it is not a *coercive* strategy but rather a *predatory* strategy. This is not to say that some strategies used by terrorist groups are not aimed at bargaining (e.g., hostage-taking, hijacking); indeed, bargaining aggression may cooccur with predatory aggression depending on the broader goals and motivations of the involved agents.

It is also interesting to consider how the use of the terms “terrorism” or “terrorist” by national governments and terrorist groups themselves can be seen as an attempt to evoke the CPT. As Sederberg (1989, p. 3) points out, this tactic serves the interests of those defining and using the term. For instance, by framing opponents as terrorists, governments can delegitimize them, portraying them as more threatening and less open to negotiation. This strategic use of language taps into the CPT, influencing public perception by making opponents seem more threatening and less open to bargaining and concessions (see Baele et al., 2019). While a full theoretical sketch is beyond the scope of the current discussion, if the CPT exists, like other threat detection systems, it is probably vulnerable to manipulation (Haselton & Buss, 2000; Sperber, 1994; Tooby & Cosmides, 2006). Manipulators may exploit pre-existing adaptive preferences in receivers to induce out-of-context behaviors by leveraging the receiver's adaptive responses, i.e., “sensory traps” (Soler et al., 2014). Therefore, further research should explore the role of deception (and signaling theory) as it relates to mass violence and terrorism (Dawkins & Krebs, 1978; Grafen, 1990; Krebs & Dawkins, 1984).

### Future Directions for Research and Policy

Several hypotheses emerge from the model presented in this paper, offering avenues for further exploration and empirical validation. [H1] *The influence of coalitional cues on terrorism labeling*: all else being equal, individuals are more likely to label violent acts as terrorism when these acts exhibit cues consistent with the CPT. [H2] *Predatory versus manipulative goals in terrorism:* attack cues indicative of predatory aggression will be less likely to result in political concessions than acts involving cues indicative of bargaining aggression. This challenges prevailing notions about the objectives of terrorism, suggesting that a reevaluation of the underlying motivations of terrorist groups could illuminate why certain strategies are met with success or failure in achieving political aims. [H3] *Differential responses to lone actor and terrorist events:* responses to acts of violence (e.g., fear, anxiety, media consumption) will exhibit notable differences when comparing lone actor to coalition-based attacks. For instance, events tapping into the “coalitional” element of the template should evoke greater and more sustained anxiety and fear than lone-actor events. There is some evidence of systematic responses to terrorism, such as an increased willingness to torture terrorist suspects to obtain information but not punish them blindly and for increased memory of terrorist suspects (Lindner, 2018). However, to the author's knowledge, no studies currently examine the potential of such nuanced differential reactions to lone actor versus coalitional violence (however, see Campbell-Obaid & Lacasse, 2023). Indeed, precautionary responses are expected to be tailored to the nature of the threat faced (Boyer & Liénard, 2006). Moving forward, validating such hypotheses could significantly refine our understanding of terrorism and ensure that responses are calibrated to the realities of the threats faced, rather than misaligned perceptions.

The current study underscores the inherent difficulties in defining terrorism. Individual differences and varied responses to terrorism could be key factors contributing to the legal ambiguity surrounding terrorism. This is apparent when compared to the clearer legal conception of genocide (O’Brien, 2020), a situation of violence closely aligned with the input conditions the CPT evolved to detect and respond to. It also underscores the often-overlooked role of coordination problems in legal contexts (see McAdams, 2009).

Furthermore, the model urges a reevaluation of our responses to terrorism. In highly cooperative societies, there is a tendency toward responding to terrorism with a subordinate and conciliatory rationality (Tooby, 2015). Subordinate rationalities are ill-suited to confront predatory rationalities that interpret such responses as weaknesses to be exploited and opportunities for intensifying aggression (Tooby, 2015). However, in contrast, responding to an attacker's predatory rationality with a similarly aggressive, indiscriminate, and unrestrained approach risks conflict escalation and the violation of international human rights law or international humanitarian law (e.g., Aceves, 2020; De Graaf, 2011; Parker, 2019). Acknowledging the impact of the CPT on our perception of terrorism may pave the way for more innovative and balanced responses. These approaches should aim to counteract the predatory rationality underlying many acts of violence while also preventing the appeasement of terrorist motives. Establishing clear behavioral boundaries and legal expectations (making focal the form of behavior the law demands)^8^ coupled with frameworks for effective coordination (see McAdams, 2009) may be effective legal strategies. Policymakers are encouraged to reexamine the role of deterrence in countering terrorism, ensuring that such strategies are informed by an understanding of our cooperative rationality compared to the oft-predatory rationality of potential attackers (Thayer, 2007).

## Notes

1 A coalition is an *n*-person coordination for achieving shared militant goals.

2 This does not suggest that the CPT is a physical or localized part of the mind/brain. Rather, humans possess a representational template [PREDATORY + COALITION] that downstream systems can interpret and act upon (see section “Evolved Mechanisms for Threat Detection”).

3 The observation that such definitions may be more reflective of the preferences or commitments of their proposers rather than mirroring real-world conditions is an important consideration (I. Sobol, personal communication, November 24, 2023). Nevertheless, it is intriguing to note that even within legal definitions, coalitional elements persistently emerge.

4 The recalibrational theory of anger posits that anger functions to adjust the behavior of individuals who undervalue the welfare of the person expressing the anger in their decision-making (Delton & Robertson, 2016; Sell, 2011; Sell et al., 2017). The concept of welfare tradeoff ratio (WTR) refers to how an individual weighs her welfare against that of others in making decisions in situations of cooperation and conflict (Delton & Robertson, 2016; Sell et al., 2009; Tooby et al., 2008). Essentially, anger is a bargaining tool to increase the target's WTR in favor of the angered in future interactions. As an internal regulatory variable, the WTR intervenes in the specification of motivations, emotions, and decisions to act in particular ways. A full discussion of this theory is beyond the scope of this paper, but see Tooby et al. (2008).

5 Lone perpetrators may indeed have higher rates of mental illness (e.g., Gruenewald et al., 2013; Spaaij, 2010) and such correlations may partially account for such attributions. However, this effect may not entirely account for how one's coalitional identity seems to turn “irrational” acts of violence into “rational” ones despite the nature of the attack remaining the same.

6 It may be easier to attribute a “rational” motivation to an outgroup member in conflict with your in-group as it is readily apparent why the enemy wants to kill you—they are your *enemy*! However, the logic of in-group violence is more ambiguous (M. Landers, personal communication, December 06, 2023). This is not to say that participants will not appeal to explanations for the behavior that more closely matches the cooperative rationality to which they are accustomed; for instance, the violence is driven by grievance or desperation (Moncrieff & Lienard, 2023; Tooby, 2015).

7 As a reviewer noted, it is possible that populations that evolved under the threat of frequent conflicts, where attacks are closely followed by others (high temporal autocorrelation), are likely to assess future risks differently from populations where conflicts were sporadic and attackers’ resources were quickly depleted (negative temporal autocorrelation). Depending upon conditions, the immediate response to an attack would have been critical as under-reacting would have increased the risk of subsequent attacks. In contrast, a strong and decisive response could potentially deter future threats. Obtaining reliable estimates of raids and wars in our ancestral past is challenging, let alone determining differential impacts on specific populations. Existing data suggest that mortality rates from intergroup conflict were high enough to exert significant evolutionary pressure on social behaviors (Bowles, 2009), and further research and modeling may help uncover more nuanced patterns. Nonetheless, research is still limited regarding how historical patterns of coalitional predation have influenced contemporary responses to such threats across different populations.

8 The norms surrounding language used at airports vividly illustrate clearly defined actions that are restricted in the context of security and counterterrorism. For instance, there is a societal and legal expectation to refrain from mentioning explosives while in an airport. This norm is not merely a social construct but is reinforced by legal consequences, making it a clear, focal behavior that the law restricts. Such focal behaviors, though sometimes appearing trivial or overly cautious, may play a significant role in security and counterterrorism efforts.
